# Correlation between Solubility Parameters and Properties of Alkali Lignin/PVA Composites

**DOI:** 10.3390/polym10030290

**Published:** 2018-03-08

**Authors:** Gaofeng Zhao, Haiyue Ni, Shixue Ren, Guizhen Fang

**Affiliations:** Material Science and Engineering College, Key Laboratory of Bio-Based Material Science and Technology Ministry of Education, Northeast Forestry University, Harbin 150040, China; zgf1993@yeah.net (G.Z.); 18766566501@163.com (H.N.)

**Keywords:** alkali lignin, composite membrane, inverse gas chromatography, the solubility parameter, preparation of composites, performance of composites

## Abstract

Although lignin blending with thermoplastic polymers has been widely studied, the usefulness of the lignin–polymer composites is limited by the poor compatibility of the two components. In the present study, alkali lignin/PVA composite membranes were prepared by incorporating 10%, 15%, 20% and 25% alkali lignin into the composites. The thermodynamic parameters of the composites were measured using inverse gas chromatography (IGC). Composite membranes with 10%, 15%, 20%, and 25% alkali lignin had solubility parameters of 17.51, 18.70, 16.64 and 16.38 (J/cm^3^)^0.5^, respectively, indicating that the solubility parameter firstly increased, and then decreased, with increasing proportions of alkali lignin. When the alkali lignin content was 15%, the composites had the largest solubility parameters. The composite membrane with an alkali lignin content of 15% had a tensile strength of 18.86 MPa and a hydrophilic contact angle of 89°. We have shown that the solubility parameters of blends were related to mechanical and hydrophilic properties of the composites and the relationships have been verified experimentally and theoretically.

## 1. Introduction

Apart from cellulose, lignin is the most abundant macromolecule and is the only large-volume renewable feedstock that is composed of aromatics [1]. Lignin regenerates quickly per year, and alkali lignin is available in large quantities from numerous pulping processes in China [2,3]. Lignin, which is a complex three-dimensional network rich in active functional groups, including phenols and methoxy groups, has the advantages of being nontoxic, renewable, and biodegradable [4,5]. Lignin also contains abundant phenylpropane units which are relatively hydrophobic and aromatic in nature [6]. Much attention has been paid to lignin as a globally available biomass and various compositions have been subjected to surface modifications to modify their original properties [7]. Lignin has been used in composites with plastic and rubber or as an additive of adhesives to produce novel polymers with antimicrobial activity, low toxicity, and good resistance to weathering and ultraviolet irradiation [8,9,10,11]. Lignin blending with polymer composites has become a feasible way to research high-value utilization of lignin.

Alkali lignin is a co-product of biofuel production and the paper industry and is also the most common type of lignin that is produced [12]. Polyvinyl alcohol (PVA), a polymer that has good film-forming, mechanical, and compatibility properties, contains a large number of hydroxyl groups, which confer high polarity and aqueous solubility [13]. Composite materials comprising alkali lignin and PVA held together by intermolecular forces can be prepared by simple mechanical blending [14]. Although interfacial compatibility is good, these composites still show obvious phase separation. The solubility parameter is widely used for predicting compatibility between two materials [15,16,17,18]. Materials with similar solubility parameter will show physical affinities, so lignin forms miscible blends with polyethylene terephthalate (PET) and polyethylene oxide (PEO) and immiscible blends with polypropylene (PP) and polyvinyl alcohol (PVA) [19].Compatibility of the composite interface is usually evaluated by analyzing chemical interactions, and measuring thermal and mechanical properties, or by the swelling method, which allows qualitative analysis of two-component interactions [20,21]. Deshpande et al. [22] were among the first to apply an inverse gas chromatography (IGC) technique for the determination of the interaction between a polymer and a nonpolymeric compound. Later, this method was also utilized to measure the compatibility of polymer blends [23,24,25]. Quantitative characterization of the composite of PVA and alkali lignin is, however, lacking and few studies have provided a quantitative representation of the interactions between the two components and the relationship with their properties.

Inverse gas chromatography (IGC) has been used to determine the physicochemical properties of polymers in organic materials such as powders [26], crude oils [27], nanomaterials [28], fibers [29], copolymers [30], polymer blends [31], hyperbranched polymers [32], and some nonfood carbohydrates [33]. IGC has also been used to determinate the solubility parameter of polymers [34,35]. The technique requires a chromatographic column filled with the material under study [36]. A series of probe solvents and test temperatures are selected, and the retention volume ($V_{g}^{0}$) is used to quantify the interaction of the probe solvent with the stationary phase at the working temperature.

The present study attempts to introduce solubility parameter theory into a quantitative study of the compatibility of lignin composite materials. The solubility parameters and related thermodynamic parameter of alkali lignin/PVA were quantified by IGC and the relationship between solubility parameters and relevant properties of composites was established.

## 2. Materials and Methods

### 2.1. Materials

Commercial alkali lignin, isolated by alkali-assisted extractions from wheat straw (composition: 82.69% lignin, 8.35% carbohydrates, and 8.96% ash) was supplied by Tralin Paper Co., Ltd. (Shandong, China). PVA 2488 (AR) was supplied by Sinopec Sichuan Vinylon Works (Changshou, China). Organic solvents were obtained from Aladdin Industrial Co., Ltd. (Shanghai, China).

### 2.2. Determination of Solubility Parameters of Alkali Lignin/PVA Composite Materials by IGC

The solubility parameter was measured using an Agilent 6890 N Gas Chromatograph (Agilent Technologies, Beijing, China). Mixtures of PVA and alkali lignin and 6201 red diatomaceous earth (1:10 *w*/*w*) were uniformly mixed with moderate acetone and then were dried. The blends were then packed in a solvent-rinsed stainless steel column by using a mechanical vibrator and a vacuum pump. After packing, the column was conditioned overnight in a stream of nitrogen at 130 °C. Probe solvents were injected manually using a 1 µL Hamilton syringe, with column temperatures maintained at 110, 120, 130, 140, and 150 °C. To achieve infinite dilution, the injection volume for each probe solvent was 0.5 µL. At least three injections were made for each probe solvent, and the average retention time, *t*_R_, was used for the calculations of solubility parameters of alkali lignin/PVA composites.

### 2.3. Preparation of Alkali Lignin/PVA Composite Membrane

**Figure 1:**
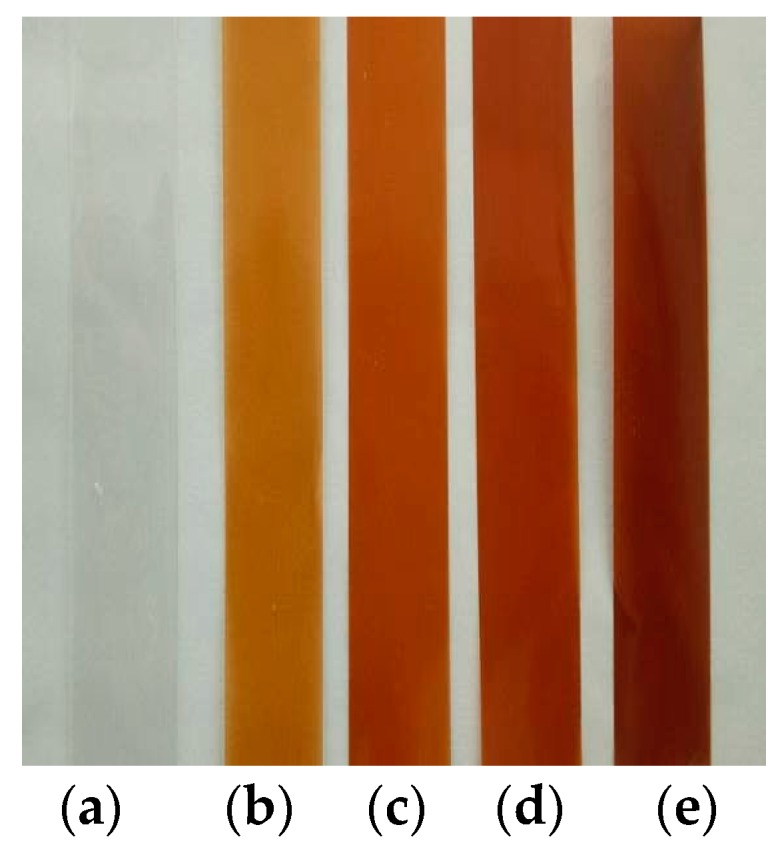
The shape of the composite materials. (**a**) PVA; (**b**) 10% alkali lignin/PVA; (**c**) 15% alkali lignin/PVA; (**d**) 20% alkali lignin/PVA; (**e**) 25% alkali lignin/PVA.

Blends of PVA and 10%, 15%, 20%, and 25% alkali lignin were placed in a 250 mL three-necked flask and stirred at the speed of 500 r/min for about 2 h in a water bath at 90 °C. After ultrasound treatment for 20 min, bubbles were removed in a vacuum oven (0.06 MPa). The solution was spread onto a film plate and dried for 24 h at room temperature to form a homogeneous film, with a thickness of approximately 60–80 µm. The composite material shapes were cut in a strip shape (as shown in Figure 1).

### 2.4. Test of Mechanical Properties of Alkali Lignin/PVA Composite Membranes

The mechanical properties of the alkali lignin/PVA composite membranes were measured using a universal testing machine (LDX-200, Beijing Landmark Packaging Material Co., Ltd., Beijing, China). To determine the average thickness of the sample mold, the thickness at 10 randomly chosen points on the sample was measured using a screw micrometer. Five rectangular strips, 160 mm × 20 mm, were cut from each sample according to the GB/T13022-1991 standard and the composite membranes were treated according to the GB/T2918-1998 standard. After treatment, samples were moved into a container with relative humidity of 50% for about 90 h. The tensile strength of the composite membranes was measured using a universal testing machine at a speed of 50 mm/min and the average value from five measurements was recorded for each sample.

### 2.5. Test of Hydrophilic Properties of Alkali Lignin/PVA Composite Membranes

The water contact angle is a measure of the intrinsic hydrophilicity and the surface wettability of the material [37]. The contact angle of the membrane surface was measured using a static contact angle meter (JC2000C, Shanghai Zhongchen Digital Technic Apparatus Co., Ltd., Shanghai, China). The smooth section of the alkali lignin/PVA composite membrane was cut into a shape 15 mm × 15 mm, with three samples for each group. The sample was placed on the experimental platform of the contact angle meter at room temperature and a drop of deionized water (5 μL) was then dripped on the surface of the sample. At a set time (10 s), the membrane was photographed and the contact angle of the sample membrane was measured. Each sample was measured three times and the average value was used in the calculations.

## 3. Results and Discussion

### 3.1. Solubility Parameters of Alkali Lignin/PVA Composite Membranes with Different Proportions of Alkali Lignin

#### 3.1.1. Retention Volumes of Probe Solvents

The retention volume, $V_{g}^{0}$, was calculated using Equation (1):

(1) $$V_{g}^{0}=273.15JF\frac{\Delta t}{mT}$$

where ∆*t* = *t_r_* − *t_m_*, *t_r_* is the retention time of the adsorbing solute probes, *t_m_* is the mobile phase (n-pentane) hold-up time (dead time), *F* is the flow rate under ambient conditions, m is the mass of the solvent on the column packing, and *T* is the column temperature (K). The factor *J*, which corrects for the influence of the pressure drop along the column, is given by

(2) $$J=\frac{3}{2}\frac{{\left(P_{i}/P_{0}\right)}^{2}-1}{{\left(P_{i}/P_{0}\right)}^{3}-1}$$

where *P_i_* and *P*_0_ are the inlet and outlet pressure, respectively [38].

As shown in Table A1 (Appendix A), alkali lignin and PVA are both polar and, according to the theory of similarity and compatibility, the proper proportion of alkali lignin in a polar polymer results in good interfacial compatibility of the polymer composite. At a given temperature, $V_{g}^{0}$ increases as the amount of alkali lignin in the composite increases. Because of its unique three-dimensional network structure, when the amount of alkali lignin increases, the small molecules of the probe solvent are more easily introduced into the polymer, resulting in stronger interactive forces between the two. As the strength of the interaction between the probe solvent and the polymer increases, the retention time of the probe solvent in the column increases and the value of $V_{g}^{0}$ for the probe solvent also increases.

#### 3.1.2. Thermodynamics Parameters of Probe Solvents

The weight fraction activity coefficient of the probe solvent at infinite dilution, $\Omega_{1}^{\infty }$, was calculated using Equation (3):

(3) $$\ln\Omega_{1}^{\infty }=\ln\frac{273.15R}{P_{1}^{0}V_{g}^{0}M_{1}}-\frac{P_{1}^{0}}{RT}(B_{11}-V_{1})$$

where *M*_1_ is the molecular mass of the probe solvent, *R* is the ideal gas constant, *B*_11_ is the second virial coefficient, $P_{1}^{0}$ is the saturated vapor pressure, and *V*_1_ is the molar volume of the probe solvent. The values of *B*_11_, $P_{1}^{0}$, and *V*_1_ were calculated at column temperature [38].

The molar absorption enthalpy, Δ$H_{1}^{s}$, was obtained from the slope of the plot of 1/*T* versus ln$V_{g}^{0}$ in Equation (4):

(4) $$\Delta H_{1}^{s}=-R\frac{\partial (\ln V_{g}^{0})}{\partial (1/T)}.$$

The partial molar heat of mixing, Δ$H_{1}^{\infty }$, was obtained from the slope of the plot of 1/*T* versus ln $\Omega_{1}^{\infty }$ in Equation (5):

(5) $$\Delta H_{1}^{\infty }=R\frac{\partial (\ln\Omega_{1}^{\infty })}{\partial (1/T)}.$$

The molar evaporation enthalpy, Δ*H_v_*, of the probe solvent adsorbed by the polymers is related to Δ$H_{1}^{\infty }$ and Δ$H_{1}^{s}$ as follows:

(6) $$\Delta H_{v}=\Delta H_{1}^{\infty }-\Delta H_{1}^{s}.$$

As is shown in Table A2 (Appendix A), values of Δ$H_{1}^{s}$ for all of the probe solvents were negative, indicating that adsorption of the probe solvent onto the polymer is an exothermic process. Values of Δ*H_v_* for all of the probe solvents were positive, indicating that evaporation of the probe solvent from the polymer is an endothermic process. As the alkali lignin content increased, the absolute value of Δ$H_{1}^{s}$ firstly increased and then decreased.

#### 3.1.3. Interaction Parameters

According to the Flory–Huggins theory, the interaction parameter, $\chi_{12}^{\infty }$, of a given solute–polymer pair is defined as [38]

(7) $$\chi_{12}^{\infty }=\ln\frac{273.15R}{P_{1}^{0}V_{g}^{0}M_{1}}-\frac{P_{1}^{0}}{RT}(B_{11}-V_{1})-1.$$

As shown in Table A3 (Appendix A), when the value of $\chi_{12}^{\infty }$ is <0.5 (critical value), the probe solvent is generally characterized as a good solvent, whereas a value >1 designates a poor solvent that may lead to phase separation [26]. Because PVA is insoluble in these probe solvents, all values of $\chi_{12}^{\infty }$ are >1 when only a small amount of alkali lignin was added. When the proportion of alkali lignin reached 25%, values of $\chi_{12}^{\infty }$ for all probe solvents, except tetrahydrofuran, acetone and methyl ethyl ketone, were >1. The values of $\chi_{12}^{\infty }$ that are <1 are attributable to the solubility of alkali lignin, and the values reflect the inherent properties of PVA and alkali lignin.

#### 3.1.4. Solubility Parameters

The solubility parameter of each probe solvent, *δ*_1_, was calculated using Equation 8:

(8) $$\delta_{1}={\left(\frac{\Delta E_{v}}{V_{1}}\right)}^{\frac{1}{2}}={\left(\frac{\Delta H_{v}-RT}{V_{1}}\right)}^{\frac{1}{2}}$$

where Δ*E_v_* is the energy of vaporization of the compound, *V*_1_ is the molar volume of the compound, and Δ*H_v_* is the molar heat of vaporization of the compound.

Values of *δ*_1_ decreased with increasing temperature for two reasons: (i) the heat of vaporization decreases with temperature; and (ii) the molar volume increases with temperature. The solubility parameter of the polymer, *δ*_2_, was calculated using Equations (9) and (10):

(9) $$\left(\frac{\delta_{1}{}^{2}}{RT}-\frac{\chi_{12}^{\infty }}{V_{1}}\right)=\left(\frac{2\delta_{2}}{RT}\right)\delta_{1}-\left(\frac{\delta_{2}{}^{2}}{RT}+\frac{\chi_{s}^{\infty }}{V_{1}}\right),$$

(10) $$\delta_{2}=\frac{kRT}{2},$$

where $\chi_{s}^{\infty }$ is the entropy term of the Flory–Huggins interaction parameter and *k* is the slope of Equation (9) [35,39].

**Figure 2:**
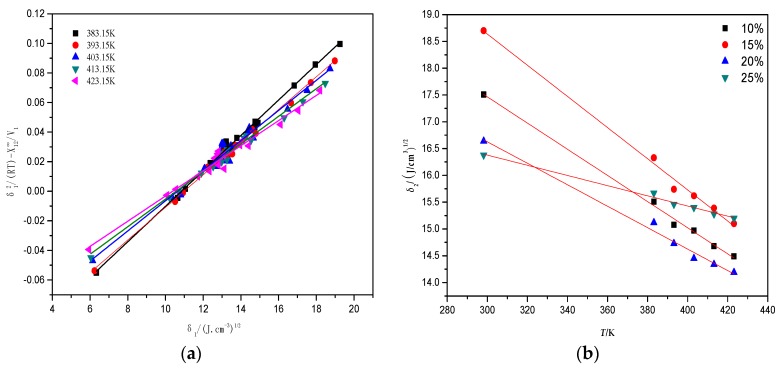
(**a**) The calculated example of the solubility parameter (PVA); (**b**) Solubility parameters of different proportions under 298.15 K by the extrapolation method.

According to Equation (9), the correlation of *δ*_1_^2^/(RT)-$\chi_{12}^{\infty }$/*V*_1_ and *δ*_1_ was obtained as shown in Figure 2a. The different slopes can be obtained, and values of *δ*_2_ for the alkali lignin/PVA composite membrane at 383, 393, 403, 413 and 423 K are shown in Table 1 according to Equation (10). As calculated by the extrapolation method [35] (Figure 2b), values of *δ*_2_ at 298.15 K for alkali lignin/PVA composite membranes with alkali lignin contents of 10%, 15%, 20%, and 25% were 17.51, 18.70, 16.64, and 16.38 (J/cm^3^)^0.5^, respectively.

### 3.2. Relationship between Mechanical Properties and Solubility Parameters (δ*_2_*) of Lignin/PVA Composite Membranes

**Figure 3:**
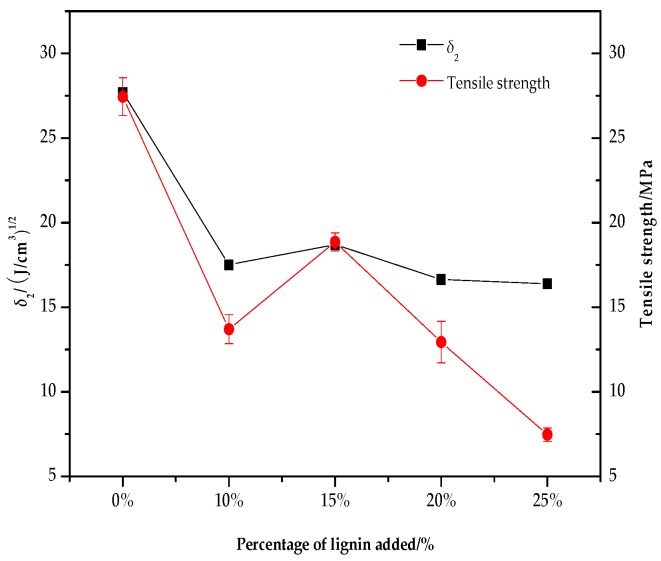
Effect of different proportions of alkali lignin on *δ*_2_ and tensile strength.

**Figure 4:**
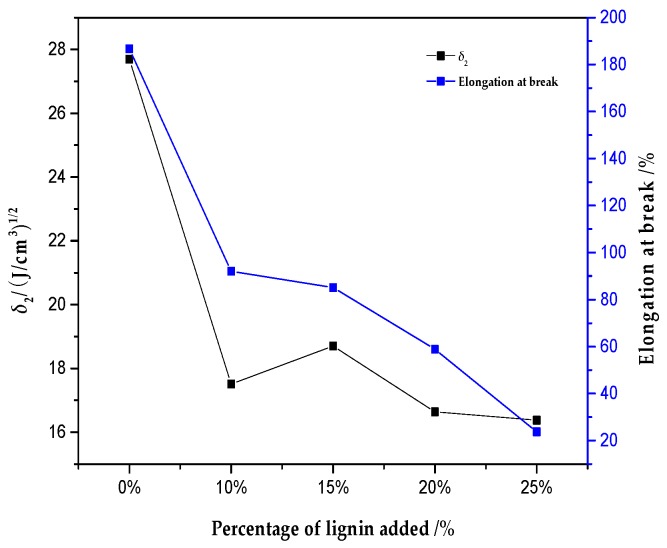
Effect of different proportions of alkali lignin on *δ*_2_ and elongation at break.

The relationship between the solubility parameter (*δ*_2_) and the tensile strength and elongation at the break of the lignin/PVA composite film is shown in Figure 3 and Figure 4. As the proportion of alkali lignin increases, the solubility parameter and tensile strength of the composites firstly decreases, then increases, and finally decreases again. When the alkali lignin content was 10% or 15%, two peaks appeared. The difference between the two values of the solubility parameter of alkali lignin (*δ* = 20.09 (J/cm^3^)^0.5^) and PVA (*δ* = 27.69 (J/cm^3^)^0.5^) is large, which might lead to bad compatibility. When alkali lignin was added (10%), the single system might be broken and both the solubility parameter and tensile strength decreased. With added alkali lignin (15%), their interaction may increase because of hydrogen bond function increasing compatibility. More alkali lignin was added (20% and 25% lignin), leading to the internal structure of composites being loose and, consequently, bad compatibility. As the proportion of alkali lignin increases, elongation at the break decreases continuously, which may be partly due to the fact that alkali lignin and PVA chains can bind tightly, but also partly due to the fact that alkali lignin only acts as infilling in the network structure. Also, it can be concluded that the reason for the reduction of elongation at the break of the blend membranes may be due to the low ductility of membranes as the mass of alkali lignin increases.

**Figure A1:**
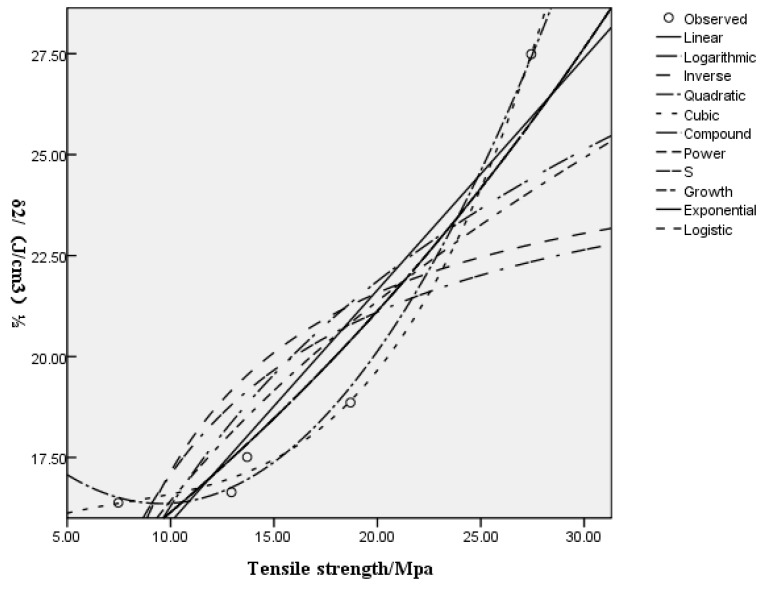
Model summary between tensile strength and the solubility parameter.

The solubility parameters (*δ*_2_) and related thermodynamic parameters of alkali lignin/PVA composite membranes were measured by IGC and were analyzed at different temperatures. The tensile strength of the composite membranes was measured using a universal testing machine and the relationship between the solubility parameter and mechanical strength was also analyzed. These experiments were designed to establish a quantitative relationship between the solubility parameter (*δ*_2_) and the tensile strength of the alkali lignin/PVA composite membrane, and to provide a reference for study of the relationship between the interface compatibility and the mechanical strength of alkali lignin/PVA composites (Figure A1). The degree of difference in the linear regression equation was tested using the F-test. The binomial relationship between the solubility parameter and the tensile strength of the lignin/PVA composite membrane was calculated using the F-test. Values of *F* = 171.868 > *F* (1, 3) = 10.128 (Appendix C) and *p* = 0.006 < 0.05 (Table A4) were obtained, indicating that X impacts Y significantly and R^2^ has statistical significance by the statistical method, meeting the trend of the quadratic function. Therefore, there is a significant correlation between the solubility parameter and the tensile strength of the composite.

**Figure 5:**
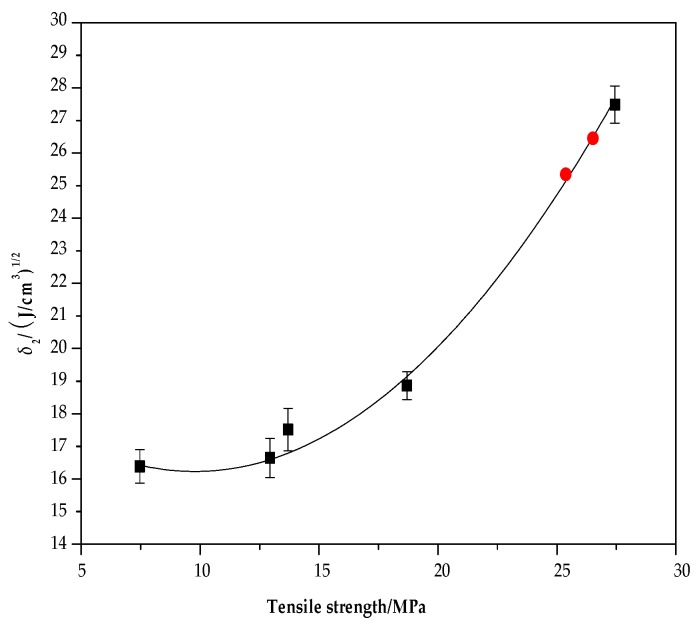
Tensile strength vs solubility parameter (*δ*_2_) of alkali lignin/PVA composite membranes.

The relationship between the tensile strength and the solubility parameter (*δ*_2_) of the alkali lignin/PVA composite membranes is shown in Figure 5. The relationship between the two parameters, obtained by mathematical analysis, fitted the binomial relationship *Y* = 19.797 − 0.709*X* + 0.036*X*^2^ (0 < *X* < 27.44), where *X* is the tensile strength and *Y* is the solubility parameter of the composite. The linear correlation coefficient was 0.989. The tensile strength of lignin/PVA composites increased with increases in the value of the solubility parameter.

To verify the reliability of the model, lignin/PVA composite membranes with alkali lignin proportions of 3% and 5% were measured by the same method. The solubility parameters of composite membranes with alkali lignin contents of 3% and 5% were 26.29 and 25.16 J/cm^3^, respectively, and the values of tensile strength were 26.40 and 25.30 MPa, respectively (Figure 5). The solubility parameters, calculated using the binomial relationship, were 26.36 and 25.09 J/cm^3^, indicating that the theoretical value obtained from the binomial was consistent with the actual measured value. The tensile strength of lignin/PVA composite membranes increased as the solubility parameter increased.

### 3.3. Relationship between Contact Angle and Solubility Parameter (δ*_2_*) of Alkali Lignin/PVA Composite Membranes

**Figure 6:**
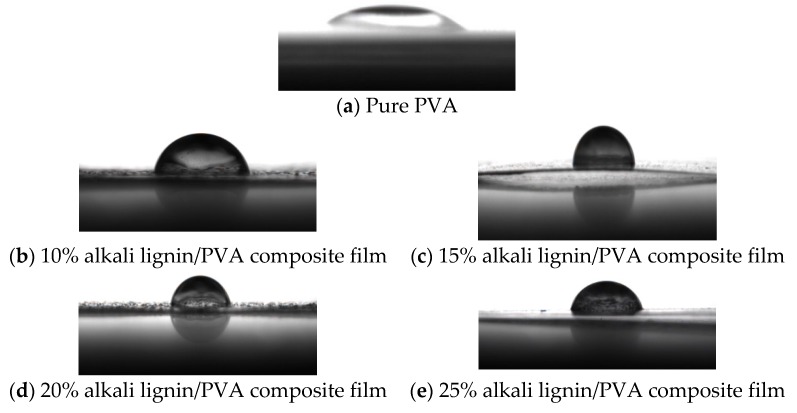
Contact angles of alkali lignin /PVA composite membranes.

The contact angles of the alkali lignin/PVA composite membranes were measured on a static contact angle meter, using 5 μL of deionized water at room temperature. Each sample was measured three times and average values were calculated. The contact angles of alkali lignin/PVA composite membranes with alkali lignin contents of 0%, 10%, 15%, 20%, and 25% were 34.5°, 77.0°, 89.0°, 79.0°, and 74.5°, respectively. PVA contains a large number of hydroxyl groups and, when dissolved in aqueous solution, these form hydrogen bonds with water, so the PVA film contact angle is small (Figure 6a). Because of the low hydrophilicity of alkali lignin, the contact angle of the membrane firstly increased markedly when alkali lignin was added. The contact angle of the membrane decreased because of poor compatibility as the proportion of alkali lignin increased further (Figure 6). This can be explained by the internal structure of composites being loose, and the fact that water molecules easily penetrated the composites above 15% alkali lignin. The contact angle was largest when the proportion of alkali lignin was 15%, indicating that this membrane has good hydrophobicity. The hydrophobicity of the membrane is consistent with the maximum value of *δ*_2_ obtained for the 15% alkali lignin/PVA composite membrane.

**Figure 7:**
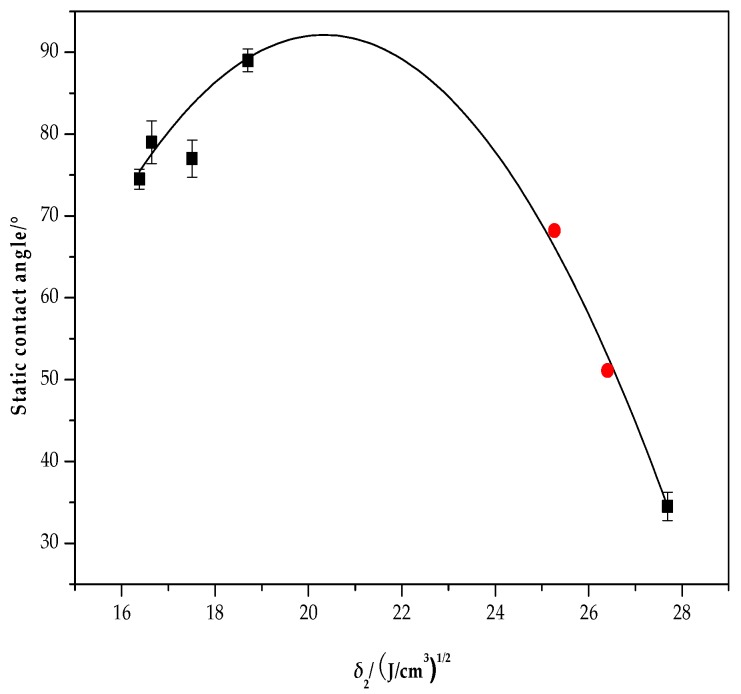
Contact angle vs the solubility parameter (*δ*_2_) of alkali lignin/PVA composite membranes.

**Figure A2:**
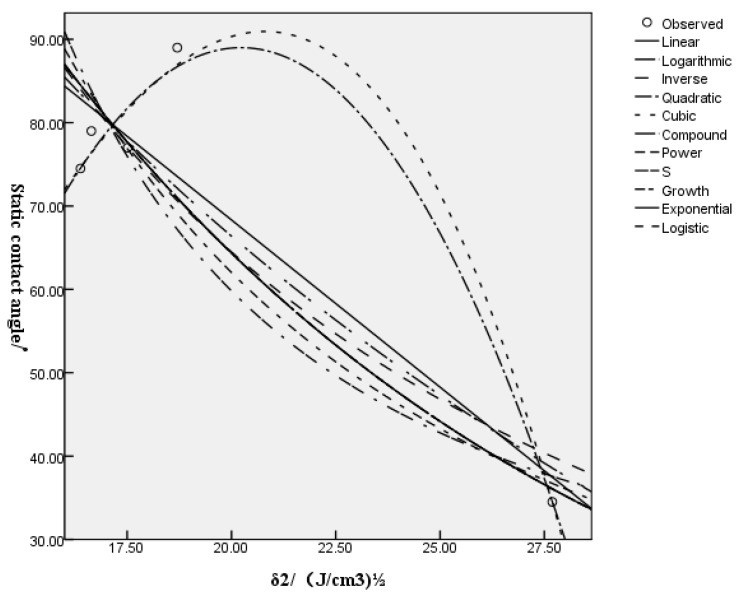
Model summary between the solubility parameter and contact angle.

A quantitative relationship between the solubility parameter (*δ*_2_) of the alkali lignin/PVA composite membrane and the static contact angle of the surface of the film has thus been established and provides a reference for study of the relationship between the lignin/PVA composite interface and its contact angle (Figure A2). The binary relationship of the solubility parameter and the static contact angle of the composite membrane was tested by the regression equation F-test, *F* = 87.579 > *F* (1, 3) = 10.128 and *p* = 0.01 < 0.05 (Table A5), indicating that X impacts Y significantly and *R*^2^ has statistical significance by the statistical method, meeting the trend of the quadratic function. Therefore, there is a significant correlation between the solubility parameters and the static contact angle of the composite membrane. The relationship between the static contact angle and the solubility parameter (*δ*_2_) of alkali lignin/PVA composite membrane is shown in Figure 7. The binomial relationship *Y* = −343.258 + 42.650*X* − 1.048*X*^2^ (16.38 < *X* < 27.69) was obtained through mathematical analysis, where *X* is the solubility parameter and *Y* is the static contact angle of the composite membrane. The linear correlation coefficient is 0.977. The static contact angle of the film firstly increases and then decreases as the solubility parameter (*δ*_2_) of the composite membrane increases. The maximum static contact angle occurs when the solubility parameter is between 20 and 21 (J/cm^3^)^0.5^.

To verify the reliability of the model, the static contact angle of alkali lignin/PVA composite membranes with alkali lignin contents of 3% and 5% were measured using the same method; the values of the static contact angle were 51.1° and 68.0°, respectively. The static contact angles calculated using the binomial relationship were 52.1°, and 65.3°, indicating that the theoretical value obtained from the binomial was consistent with the actual measured value. The static contact angle of the membrane was shown to firstly increase and then decrease as the solubility parameter (*δ*_2_) of the composite membrane increases.

## 4. Conclusions

The aim of this study was to quantitatively evaluate the compatibility of alkali lignin/PVA composites. Solubility parameters and thermodynamic parameters were determined by IGC. When the proportion of alkali lignin was 25%, alkali lignin/PVA composites dissolved in the probe solvent of tetrahydrofuran under the range of conditions used in this study. When the proportion of alkali lignin was 15%, the composite had the highest solubility parameter (18.70 (J/cm^3^)^0.5^) and the composite had the best tensile strength (18.86 MPa) and hydrophilicity (contact angle 89°). We also established a relationship between the solubility parameter (*δ*_2_) and tensile strength, which fitted the binomial relationship *Y* = 19.797 − 0.709*X* + 0.036*X*^2^ (0 < *X* < 27.44), where *X* is the composite material solubility parameter and *Y* is the composite tensile strength. A relationship between the solubility parameter (*δ*_2_) and the tensile strength was also established and fitted the binomial relationship *Y* = −343.258 + 42.650*X* − 1.048*X*^2^ (16.38 < *X* < 27.69). The polarity of PVA is higher than that of alkali lignin and the difference in the solubility parameters of PVA (27.69 (J/cm^3^)^0.5^) and alkali lignin (20.09 (J/cm^3^)^0.5^) has a bad effect on the compatibility. In future study, alkali lignin will be modified to narrow the difference between the solubility parameters of alkali lignin and PVA for better compatibility.

## Figures and Tables

**Table 1:** Solubility parameters (*δ*_2_) of the mixture at different temperatures (J/cm^3^)^0.5^.

Alkali Lignin Content	383 K	393 K	403 K	413 K	423 K
0%	19.08	18.04	17.08	16.02	14.97
10%	15.51	15.08	14.97	14.68	14.49
15%	16.33	15.74	15.62	15.39	15.04
20%	15.12	14.73	14.45	14.50	14.27
25%	15.67	15.46	15.40	15.20	15.34

## Appendix A

**Table A1:** Specific retention volumes ($V_{g}^{0}$) at different temperatures (mL·g^−1^).

**Probe Solvent**	**10%** **Alkali Lignin**					**15%** **Alkali Lignin**				
	**383 K**	**393 K**	**403 K**	**413 K**	**423 K**	**383 K**	**393 K**	**403 K**	**413 K**	**423 K**
*n*-hexane	1.01	0.68	0.4	0.38	0.31	1.13	0.82	0.62	0.21	0.32
*n*-heptane	3.03	2.11	1.35	1.09	0.79	3.83	2.47	1.72	1.12	0.8
*n*-octane	8.28	5.41	3.54	2.48	1.73	10.31	6.69	4.51	2.96	2.17
*n*-nonane	19.46	12.32	8.08	5.6	3.87	22.49	15.12	10.2	6.54	4.7
*n*-decane	41.29	25.72	16.79	11.24	7.61	49.49	31.06	21.03	13.45	9.37
cyclopentane	0.2	0.13	0.08	0.11	0.07	0.1	0.14	0.04	−0.08	0.08
cyclohexane	1.32	0.85	0.6	0.48	0.37	1.28	1.05	0.66	0.29	0.28
benzene	2.18	1.44	1.07	0.95	0.77	2.36	1.56	1.24	0.79	0.76
methylbenzene	7.14	4.33	2.84	2.32	1.64	7.76	5.5	3.84	2.37	1.73
tetrahydrofuran	3.57	2.37	1.79	1.52	1.21	3.73	2.79	2.12	1.29	1.17
methanol	8.24	5.26	3.82	2.99	2.34	12.52	8.25	6.05	4.12	3.34
ethanol	6.94	4.1	3.04	2.21	1.55	8.69	5.13	3.58	2.21	1.97
1-propanol	6.35	3.8	2.57	1.91	1.39	7.61	3.66	2.87	2.04	1.41
isopropanol	5.32	3.57	2.23	1.79	1.3	5.01	3.3	2.43	1.46	1.25
acetone	4.69	2.11	1.53	1.47	1.13	4.32	2.84	2.34	1.29	1.37
methyl ethyl ketone	5.1	3.91	3.02	2.38	1.75	5.2	4.21	3.22	2.12	1.73
methyl isobutyl ketone	13.11	10.78	7.16	6.14	4.13	14.48	10.67	8.17	5.37	4.22
dichloromethane	0.79	0.63	0.6	0.61	0.51	0.98	0.78	0.84	0.46	0.76
trichloromethane	1.68	1.31	1.03	0.93	0.8	1.91	1.65	1.37	0.87	1.09
trichloroethylene	2.99	1.99	1.43	1.28	1.02	3.29	2.38	1.72	1.29	1.17
**Probe Solvent**	**20%** **Alkali Lignin**					**25%** **Alkali Lignin**				
	**383 K**	**393 K**	**403 K**	**413 K**	**423 K**	**383 K**	**393 K**	**403 K**	**413 K**	**423 K**
*n*-hexane	1.34	0.93	0.63	0.37	0.29	2.41	2.16	1.63	1.07	0.86
*n*-heptane	3.99	2.72	1.88	1.3	0.93	7.03	5.5	4.02	2.9	2.22
*n*-octane	10.12	6.78	4.63	3.37	2.09	17.08	12.36	8.75	6.24	4.92
*n*-nonane	23.25	15.15	10.04	6.92	4.59	38.15	26.13	18.01	12.38	9.13
*n*-decane	49.07	31.89	20.64	13.9	9.36	85.77	54.61	36.02	23.09	16.61
cyclopentane	0.32	0.17	0.13	0.09	0.06	0.21	0.56	0.48	0.3	0.06
cyclohexane	1.45	1.13	0.81	0.56	0.41	2.31	2.01	1.58	1.07	0.67
benzene	2.22	1.73	1.25	0.93	0.64	3.93	3.43	2.68	2.04	1.63
methylbenzene	7.76	5.18	3.44	2.44	1.74	11.55	8.66	6.57	4.72	3.96
tetrahydrofuran	2.93	2.26	1.75	1.08	0.84	11.13	8.03	5.94	4.16	3.33
methanol	--	--	--	--	--	--	--	--	--	--
ethanol	6.17	3.65	2.53	1.91	1.4	15.59	11.43	8.71	6.05	4.69
1-propanol	4.97	3.19	2.06	1.67	1.02	15.11	10.49	7.5	5.06	3.9
isopropanol	4.41	2.99	2.06	1.39	0.93	15.43	10.53	7.23	4.91	3.73
acetone	3.28	2.19	1.5	1.2	0.76	14.39	9.99	7.54	5.3	4.15
methyl ethyl ketone	5.29	3.69	2.5	1.91	1.4	22.54	14.3	10.77	7.64	5.24
methyl isobutyl ketone	13.51	10.4	6.82	5.16	3.66	70.15	45.41	30.17	20.04	13.43
dichloromethane	0.88	0.63	0.44	0.43	0.38	1.6	1.71	1.45	0.92	0.9
trichloromethane	1.94	1.46	1.19	0.9	0.76	3.98	3.04	2.4	1.59	1.53
trichloroethylene	3.28	2.39	1.69	1.42	1.13	5.48	4.24	3.21	2.47	1.86

**Table A2:** Thermodynamic parameters of the probe solvents (kJ/mol).

**Probe Solvent**	**10%** **Alkali Lignin**			**15%** **Alkali Lignin**		
	∆$H_{1}^{s}$	∆$H_{1}^{\infty }$	∆***H_v_***	∆$H_{1}^{s}$	∆$H_{1}^{\infty }$	∆***H_v_***
*n*-hexane	−39.82	−13.66	26.16	−52.60	−26.44	26.16
*n*-heptane	−45.46	−14.79	30.67	−52.73	−22.05	30.67
*n*-octane	−52.72	−17.69	35.03	−53.05	−18.02	35.03
*n*-nonane	−54.22	−14.90	39.32	−53.48	−14.16	39.32
*n*-decane	−56.77	−13.18	43.59	−56.18	−12.59	43.59
cyclopentane	−29.11	−5.15	23.96	−12.47	11.49	23.96
cyclohexane	−42.61	−14.64	27.97	−58.03	−30.06	27.97
benzene	−33.85	−5.17	28.68	−39.66	−10.98	28.68
methylbenzene	−48.14	−15.42	32.72	−51.74	−19.02	32.72
tetrahydrofuran	−35.35	−16.11	19.24	−41.80	−22.55	19.24
methanol	−41.72	−8.09	33.63	−45.08	−11.45	33.63
ethanol	−48.78	−11.95	36.83	−51.59	−14.76	36.83
1-propanol	−50.47	−9.87	40.60	−53.58	−12.98	40.60
isopropanol	−47.48	−13.13	34.35	−48.58	−14.23	34.35
acetone	−43.69	−17.09	26.60	−41.81	−15.21	26.60
methyl ethyl ketone	−35.41	−5.82	29.59	−38.88	−9.29	29.59
methyl isobutyl ketone	−38.69	−21.42	17.27	−42.46	−25.19	17.27
dichloromethane	−12.16	12.22	24.39	−14.18	10.21	24.39
trichloromethane	−24.62	1.70	26.32	−23.99	2.33	26.32
trichloroethylene	−35.03	−4.97	30.06	−36.36	−6.30	30.06
**Probe Solvent**	**20%** **Alkali Lignin**			**25%** **Alkali Lignin**		
	∆$H_{1}^{s}$	∆***H***	∆***H_v_***	∆$H_{1}^{s}$	∆$H_{1}^{\infty }$	∆***H_v_***
*n*-hexane	−53.62	−27.46	26.16	−37.09	−10.93	26.16
*n*-heptane	−49.24	−18.57	30.67	−39.69	−9.02	30.67
*n*-octane	−51.88	−16.84	35.03	−42.77	−7.73	35.03
*n*-nonane	−54.28	−14.97	39.32	−48.63	−9.32	39.32
*n*-decane	−55.87	−12.28	43.59	−55.90	−12.31	43.59
cyclopentane	−53.73	−29.78	23.96	−40.43	−14.98	25.45
cyclohexane	−43.66	−15.69	27.97	−41.55	−13.57	27.97
benzene	−41.89	−13.21	28.68	−30.60	−1.92	28.68
methylbenzene	−50.44	−17.72	32.72	−37.06	−4.34	32.72
tetrahydrofuran	−43.41	−24.17	19.24	−41.40	−22.16	19.24
methanol	--	--	--	--	--	--
ethanol	−48.95	−12.12	36.83	−40.94	−4.10	36.83
1-propanol	−51.54	−10.94	40.60	−46.37	−5.77	40.60
isopropanol	−52.24	−17.89	34.35	−48.61	−14.25	34.35
acetone	−47.64	−21.04	26.60	−42.11	−15.52	26.60
methyl ethyl ketone	−44.82	−15.23	29.59	−47.80	−18.21	29.59
methyl isobutyl ketone	−44.61	−27.35	17.27	−55.59	−38.33	17.27
dichloromethane	−28.19	−3.80	24.39	−23.59	0.79	24.39
trichloromethane	−32.06	−5.74	26.32	−34.63	−8.31	26.32
trichloroethylene	−35.77	−5.71	30.06	−36.35	−6.29	30.06

**Table A3:** Flory–Huggins interaction parameters ($\chi_{12}^{\infty }$) of the probe solvents at different temperatures.

**Probe Solvent**	**10%** **Alkali Lignin**					**15%** **Alkali Lignin**				
	**383 K**	**393 K**	**403 K**	**413 K**	**423 K**	**383 K**	**393 K**	**403 K**	**413 K**	**423 K**
*n*-hexane	3.53	3.71	4.04	3.90	3.93	3.42	3.51	3.60	4.50	3.90
*n*-heptane	3.04	3.15	3.36	3.36	3.48	2.81	2.99	3.12	3.33	3.46
*n*-octane	2.66	2.80	2.96	3.07	3.19	2.45	2.59	2.72	2.89	2.96
*n*-nonane	2.44	2.58	2.70	2.79	2.89	2.30	2.37	2.47	2.63	2.70
*n*-decane	2.33	2.45	2.54	2.63	2.73	2.15	2.26	2.32	2.45	2.52
cyclopentane	4.85	5.12	5.40	4.87	5.16	5.57	5.04	5.99	5.19	5.06
cyclohexane	3.58	3.80	3.94	3.96	4.04	3.62	3.58	3.83	4.45	4.30
benzene	3.12	3.30	3.37	3.28	3.31	3.04	3.22	3.23	3.47	3.31
methylbenzene	2.57	2.80	2.97	2.94	3.07	2.49	2.57	2.67	2.92	3.02
tetrahydrofuran	1.21	1.46	1.59	1.62	1.73	1.17	1.30	1.42	1.78	1.76
methanol	1.95	2.12	2.19	2.19	2.21	1.53	1.68	1.73	1.87	1.86
ethanol	2.16	2.38	2.40	2.46	2.56	1.94	2.16	2.24	2.46	2.33
1-propanol	2.64	2.82	2.90	2.91	2.95	2.46	2.85	2.79	2.84	2.94
isopropanol	2.31	2.42	2.62	2.60	2.70	2.37	2.50	2.54	2.81	2.74
acetone	1.95	2.53	2.65	2.50	2.59	2.04	2.24	2.23	2.63	2.4
methyl ethyl ketone	2.29	2.31	2.34	2.37	2.48	2.27	2.24	2.28	2.48	2.49
methyl isobutyl ketone	4.43	4.47	4.74	4.78	5.07	4.33	4.48	4.61	4.91	5.05
dichloromethane	2.93	2.95	2.82	2.63	2.64	2.71	2.74	2.48	2.91	2.24
trichloromethane	2.46	2.50	2.53	2.45	2.42	2.33	2.27	2.25	2.51	2.12
trichloroethylene	2.45	2.61	2.71	2.61	2.63	2.35	2.43	2.52	2.60	2.50
**Probe Solvent**	**20%** **Alkali Lignin**					**25%** **Alkali Lignin**				
	**383 K**	**393 K**	**403 K**	**413 K**	**423 K**	**383 K**	**393 K**	**403 K**	**413 K**	**423 K**
*n*-hexane	3.24	3.39	3.59	3.93	4.00	2.66	2.55	2.63	2.86	2.91
*n*-heptane	2.77	2.90	3.04	3.19	3.31	2.20	2.19	2.27	2.38	2.44
*n*-octane	2.46	2.58	2.69	2.76	3.00	1.94	1.98	2.05	2.14	2.14
*n*-nonane	2.27	2.37	2.48	2.57	2.72	1.77	1.83	1.90	1.99	2.03
*n*-decane	2.16	2.23	2.33	2.42	2.52	1.60	1.69	1.78	1.91	1.95
cyclopentane	4.40	4.85	4.95	5.08	5.39	4.82	3.63	3.60	3.90	5.31
cyclohexane	3.49	3.51	3.63	3.81	3.93	3.02	2.94	2.96	3.15	3.43
benzene	3.10	3.11	3.22	3.31	3.49	2.52	2.43	2.45	2.52	2.55
methylbenzene	2.49	2.62	2.78	2.89	3.01	2.09	2.11	2.14	2.23	2.19
tetrahydrofuran	1.41	1.51	1.62	1.96	2.08	0.08	0.24	0.39	0.61	0.71
methanol	--	--	--	--	--	--	--	--	--	--
ethanol	2.28	2.50	2.58	2.60	2.67	1.35	1.36	1.35	1.45	1.46
1-propanol	2.88	2.99	3.12	3.04	3.26	1.77	1.80	1.83	1.93	1.92
isopropanol	2.50	2.60	2.70	2.85	3.03	1.24	1.34	1.45	1.59	1.65
acetone	2.31	2.50	2.67	2.70	2.99	0.83	0.98	1.06	1.22	1.29
methyl ethyl ketone	2.25	2.37	2.53	2.59	2.71	0.80	1.01	1.07	1.20	1.38
methyl isobutyl ketone	4.40	4.51	4.79	4.95	5.19	2.75	3.03	3.30	3.60	3.90
dichloromethane	2.82	2.95	3.13	2.97	2.94	2.22	1.95	1.93	2.21	2.07
trichloromethane	2.32	2.39	2.39	2.49	2.48	1.60	1.65	1.69	1.92	1.78
trichloroethylene	2.35	2.43	2.54	2.50	2.53	1.84	1.85	1.90	1.95	2.03

## Appendix B

**Table A4:** Model summary and parameter estimates.

Dependent Variable: *y*-Axis						
Equation	Model Summary					Parameter Estimates
	*R* Square	*F*	df1	df2	Sig.	Constant
Linear	0.866	19.396	1	3	0.022	
Logarithmic	0.696	6.856	1	3	0.079	
Inverse	0.495	2.939	1	3	0.185	
Quadratic	0.994	171.868	2	2	0.006	
Cubic	0.997	108.908	3	1	0.070	
Compound	0.892	24.847	1	3	0.016	12.339
Power	0.731	8.145	1	3	0.065	6.853
S	0.532	3.404	1	3	0.162	3.261
Growth	0.892	24.847	1	3	0.016	2.513
Exponential	0.892	24.847	1	3	0.016	12.339
Logistic	0.892	24.847	1	3	0.016	0.081

The independent variable is the *x*-axis.

**Table A5:** Model summary and parameter estimates.

Dependent Variable: *y*-Axis						
Equation	Model Summary					Parameter Estimates
	*R* Square	*F*	df1	df2	Sig.	Constant
Linear	0.815	13.217	1	3	0.036	
Logarithmic	0.779	10.548	1	3	0.048	
Inverse	0.735	8.317	1	3	0.063	
Quadratic	0.980	49.920	2	2	0.020	
Cubic	0.982	54.733	2	2	0.018	
Compound	0.876	21.286	1	3	0.019	289.986
Power	0.845	16.304	1	3	0.027	7731.486
S	0.805	12.410	1	3	0.039	2.417
Growth	0.876	21.286	1	3	0.019	5.670
Exponential	0.876	21.286	1	3	0.019	289.986
Logistic	0.876	21.286	1	3	0.019	0.003

The independent variable is the *x*-axis.

## Appendix C

There is a difference among the *n* observations and we used the observed value Y*_i_* and its mean value $\bar{Y}$, the deviation of the sum of the square, to represent the degree of difference. This is known as the total deviation of the sum of squares, and is recorded as

(A1) $$S_{\mathrm{sum}}=\overset{n}{\underset{i=1}{\sum }}{\left(Y_{i}-\bar{Y}\right)}^{2}\;=\overset{n}{\underset{i=1}{\sum }}{\left(Y_{i}-{\bar{Y}}_{i}\right)}^{2}+\overset{n}{\underset{i=1}{\sum }}{\left({\hat{Y}}_{i}-\bar{Y}\right)}^{2}$$

where $\overset{n}{\underset{i=1}{\sum }}{\left(Y_{i}-{\bar{Y}}_{i}\right)}^{2}$ represents the regression sum of squares and is expressed as S_ret_. $\overset{n}{\underset{i=1}{\sum }}{\left({\hat{Y}}_{i}-\bar{Y}\right)}^{2}$ represents the residual sum of squares and is expressed as S_res_.

(A2) $$S_{\mathrm{sum}}=S_{\mathrm{ret}}+S_{\mathrm{res}}$$

(A3) $$\begin{array}{ll} S_{\mathrm{ret}} & =\overset{n}{\underset{i=1}{\sum }}{\left({\hat{Y}}_{i}-\bar{Y}\right)}^{2}\\ & =\overset{n}{\underset{i=1}{\sum }}{\left(a+bX_{i}-a-b\bar{X}\right)}^{2}\\ & =b^{2}\overset{n}{\underset{i=1}{\sum }}{\left(X_{i}-\bar{X}\right)}^{2} \end{array}$$

The degree of freedom of S_ret_ is 1, the degree of freedom of S_res_ is *n* − 2, and the total degree of freedom is *n* − 1. If X and Y have a clear linear relationship, then

(A4) $$F=\frac{S_{\mathrm{ret}}/1}{S_{\mathrm{res}}/(n-2)}>F(1,n-2).$$
